# Ultrasonographic reference values and a simple yet practical formula for estimating average kidney length in Japanese children

**DOI:** 10.1007/s10157-022-02205-0

**Published:** 2022-04-16

**Authors:** Naoya Fujita, Osamu Uemura, Ryoko Harada, Chieko Matsumura, Tomoyuki Sakai, Yuko Hamasaki, Koichi Kamei, Kentaro Nishi, Tetsuji Kaneko, Kenji Ishikura, Yoshimitsu Gotoh, the Pediatric CKD Study Group in Japan in conjunction with the Committee of Measures for Pediatric CKD of the Japanese Society of Pediatric Nephrology

**Affiliations:** 1 Department of Pediatric Nephrology, Aichi Children’s Health and Medical Center, 426 7-chome, Morioka-cho, Obu, Aichi 474-8710 Japan; 2 Department of Pediatrics, Ichinomiya Medical Treatment and Habilitation Center, 1679-2 Tomida-nagaresuji, Ichinomiya, Aichi 494-0018 Japan; 3 Department of Nephrology, Tokyo Metropolitan Children’s Medical Center, 2-8-29 Musashidai, Fuchu, Tokyo 183-8561 Japan; 4 Department of Pediatrics, National Hospital Organization Chibahigashi National Hospital, 673 Nitonacho, Chuo-ku, Chiba, Chiba 260-8712 Japan; 5 Department of Pediatrics, Shiga University of Medical Science, Tsukinowa, Seta, Otsu, Shiga 520-2192 Japan; 6 Department of Nephrology, Toho University Faculty of Medicine, 6-11-1 Omori Nishi, Ota-ku, Tokyo, 143-8541 Japan; 7 Division of Nephrology and Rheumatology, National Center for Child Health and Development, 2-10-1 Okura, Setagaya-ku, Tokyo, 157-8535 Japan; 8 Division of Clinical Research Support Center, Tokyo Metropolitan Children’s Medical Center, 2-8-29 Musashidai, Fuchu, Tokyo 183-8561 Japan; 9 Department of Pediatrics, Kitasato University School of Medicine, 1-15-1 Kitazato, Minami-Ku, Sagamihara, Kanagawa 252-0374 Japan; 10 Department of Pediatric Nephrology, Japanese Red Cross Aichi Medical Center Nagoya Daini Hospital, 2‐9 Myoken‐cho Showa‐ku, Nagoya, Aichi 466‐8650 Japan

**Keywords:** Kidney length, Ultrasonography, Pediatric nephrology, Chronic kidney disease, Reference value, Prediction formula

## Abstract

**Background:**

The assessment of kidney size is essential for treating kidney disease. However, there are no reliable and sufficiently robust ultrasonographic reference values or prediction formulas for kidney length in Japanese children, based on a sufficient number of participants.

**Methods:**

We retrospectively analyzed kidney measurements by ultrasonography in children aged 18 years or younger from eight facilities throughout Japan between January 1991 and September 2018. Detailed reference values were developed by aggregating the left and right kidneys of boys and girls separately. Simple and practical reference values were developed by combining all the data from left and right kidneys and boys and girls. The estimation formulas for the average value and lower limit of the normal range for kidney length were developed based on regression analysis.

**Results:**

Based on the aggregated kidney length data of 1984 participants (3968 kidneys), detailed reference values and simple reference values for kidney length were determined. From the regression analysis, the formula for calculating the average kidney length was generated as “kidney length (cm) = body height (m) × 5 + 2”, and that for predicting the lower limit of normal kidney length in children under 130 cm was calculated as “lower limit (cm) = 0.85 × [body height (m) × 5 + 2]”.

**Conclusion:**

Detailed ultrasonographic reference values of kidney length for Japanese children and simple reference values and estimation formulas for daily practice have been established.

**Supplementary Information:**

The online version contains supplementary material available at 10.1007/s10157-022-02205-0.

## Introduction

Assessing kidney size is essential for treating children with kidney disease. Many kidney diseases are accompanied by changes in the morphology and size of the kidneys, and the relationship between kidney function and size in children has been shown in previous reports [1, 2]. Evaluating kidney size can also provide important insight for the diagnosis and management of chronic kidney disease. Congenital anomalies of the kidney and urinary tract in children, especially hypoplastic or dysplastic kidneys, can be determined by data like kidney size.

Ultrasonography is the most common diagnostic imaging method used to investigate kidneys and urinary tracts and can provide information on kidney size in children [3]. A reliable ultrasonographic reference value is crucial for assessing kidney size. Although some reports provided reference values for normal kidney measurements in children by ultrasonography [3–6], most of them were not reliable due to relatively small sample sizes. Therefore, it is vital to establish robust reference values for normal kidney size in children based on a large dataset. Additionally, determining a formula that could easily estimate average and lower limits of normal kidney length would be useful in clinical practice.

This study aimed to define detailed ultrasonographic reference values for kidney length in healthy Japanese children. We also tried to establish simple reference values of kidney length for daily practice and a simple yet practical formula that could estimate the normal lower limit of kidney length in children.

## Materials and methods

### Study design and data collection

In this observational retrospective study, we reviewed the medical records of pediatric participants aged 18 years or younger who underwent ultrasonography at each institution (Table 1) between January 1991 and September 2018. The inclusion criteria were as follows: (1) patients with asymptomatic hematuria, benign familial hematuria, or monosymptomatic nocturnal enuresis based on the main diagnosis at the time of ultrasonography; (2) children who underwent ultrasonography during an infant medical examination and were assumed to have normal kidneys and urinary tracts. We included participants with differences of 1 cm or more in kidney length between the left and right kidneys if there were no obvious abnormalities in morphology, internal structure, or echo intensity. We also included patients with mild hydronephrosis defined as grade 1 by the Society for Fetal Urology classification (SFU) based on the report which showed that the length of kidneys with SFU grade 1 hydronephrosis is almost equal to that of kidneys with SFU grade 0 [7].

**Table 1:** Clinical characteristics of participants and data-providing facilities

Age (years) (*n* = 1984)	8.0(4.3)
Sex	
Male	889 (44.8%)
Female	1095 (55.2%)
Body height (cm) (*n* = 1771)	124.9 (27.8)
Body weight (kg) (*n* = 1783)	28.1(14.5)
Gestational age (week) (*n* = 698)	38.9(1.7)
Birth weight (g) (*n* = 1115)	3037.1(432.4)
SFU grade	
Grade 0	1660 (83.7%)
Grade 1	303 (15.2%)
No data	21 (1.1%)
Position (*n* = 3968 kidneys)^a^	
Prone	2844 (71.7%)
Supine	1039 (26.2%)
Lateral position	73 (1.8%)
Sitting position	10 (0.3%)
No data	2 (0.05%)
The facility that provided the data for this study^b^	
Aichi Children’s Health and Medical Center	
Japanese Red Cross Aichi Medical Center Nagoya Daini Hospital	
Kitasato University School of Medicine	
National Center for Child Health and Development	
National Hospital Organization Chibahigashi National Hospital	
Shiga University of Medical Science	
Toho University Faculty of Medicine	
Tokyo Metropolitan Children’s Medical Center	

Data are presented as mean (SD) or *n* (%)

*SFU* Society for fetal urology

^a^The result is shown by the number of kidneys because there are cases in which the left and right kidneys were measured in different positions

^b^Facility names are listed in alphabetical order

The exclusion criteria were as follows: (1) patients with kidney or urological disorders (excluding asymptomatic hematuria, benign familial hematuria, and monosymptomatic nocturnal enuresis); (2) abnormal ultrasonography findings such as hydronephrosis with SFU grade 2 or higher, kidney cysts, horseshoe kidney, double pelvis, single kidney, and abnormal echo intensity; (3) patients with infectious or inflammatory diseases; (4) malformation syndrome including chromosomal abnormalities; (5) patients with a history of malignancy; (6) hypertensive patients requiring treatment; (7) patients with heart/liver/pancreatic disease requiring treatment; (8) women who were pregnant or could become pregnant; (9) participants considered inappropriate by the authors.

We obtained the following data from medical records retrospectively: date of birth, sex, date of ultrasonography examinations, kidney length (maximum longitudinal diameter) of the right and left kidneys measured by ultrasonography, body height, and body weight at the time of ultrasonography (if there was no data available on the day of ultrasonography, measurements within three months before and after the date of ultrasonography were accepted), body position at the time of ultrasonography, gestational age, birth body weight, and the presence or absence of SFU grade 1 hydronephrosis.

In this study, we only used data collected from ultrasonography results prepared by pediatric nephrologists, radiologists, and medical sonographers proficient in pediatric kidney ultrasonography. The type of ultrasound machine system, ultrasonic probe, and acoustic operating frequency were not specified.

### Reference values of kidney length for ultrasonography

Reference values of kidney length for ultrasonography for each age and body height were calculated from the collected data. Values by age were summarized as follows: every 3 months for 1 year, every 6 months between 1 and 2 years, and every year between 2 and 18 years old. Values of body height were summarized for each 10 cm body height (50 to 59.9 cm, 60 to 69.9 cm, etc.). Then, we calculated the mean ± 2 standard deviations (SD) for each age and body height group. Detailed reference value tables were created and organized separately by sex, and by right or left kidneys. Simple and practical reference value tables for daily clinical use were developed by combining all the data regardless of sex or kidney position.

### Formula for estimating kidney length by ultrasonography

Linear regression analysis was used to create a prediction formula for kidney length based on age or body height.

### A simple and practical formula for estimating kidney length by ultrasonography

A simple formula for predicting kidney length for daily clinical use was developed based on the regression formula using the combined data of all participants. To simplify the formula, we rounded off to the first decimal place for each term of the regression formula.

### A simple formula for estimating the lower limit of normal kidney length for children by ultrasonography

A formula for estimating the lower limit of normal kidney length was developed using the prediction formula for kidney length. We set the lower limit of kidney length as “mean—2 SD” and converted it to “mean × (1–2 SD/mean)” so that it could be expressed by a single equation using a coefficient and calculated “2 SD/mean” from the collected data of age or body height groups with 100 or more participants. While using this formula for screening, we adopted the rounded down values as the “2 SD/mean” so that participants with borderline values could be detected as requiring attention. To evaluate the performance of this prediction formula, we examined the rate of participants who were below the lower limit based on the lower limit value calculated from the formula. This evaluation was performed for all participants younger than 10 years of age, which is equivalent to approximately 130 cm or less in height; where kidney size is considered to be a more important indicator of congenital kidney disease than it is in older children.

### Difference in measured values at each facility

When comparing values by institution, we considered the issue of legitimacy when using a small amount of data for regression analysis, and therefore only examined institutions that reported more than 100 cases.

### Differences in measured values according to sex, kidney position, and body position

To examine the differences in kidney length according to sex, kidney position, and body position during ultrasonography, each regression line corresponding to body height and kidney length was compared to its 95% confidence intervals in all participants.

### Statistical analysis

Statistical analyses were performed using SPSS® version 26 (IBM, Chicago, USA), to clarify descriptive statistics and linear regression analysis.

## Results

### *Characteristics of the study population* (Table 1)

**Fig. 1:**
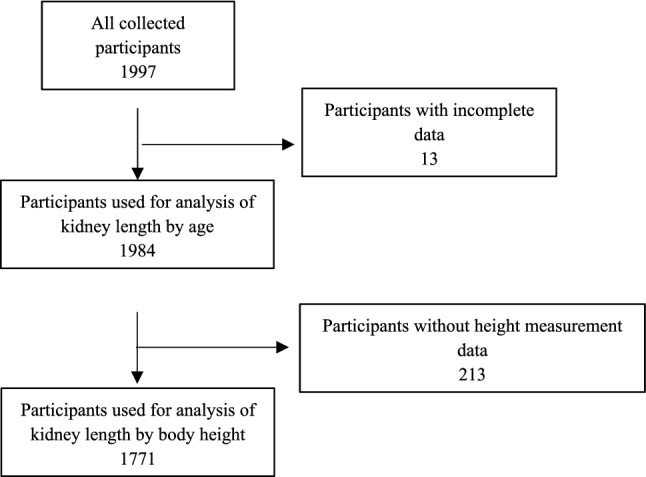
Participant selection flow chart

The data of 1997 participants were obtained from eight facilities throughout Japan. Of these, 1984 children; 889 boys and 1095 girls, who fulfilled the eligibility criteria, were included (Fig. 1). However, since height data were missing in 213 cases, 1771 (89.3%) cases were included for regression with height.

### Reference values of kidney length by ultrasonography

Tables 2 and 3 show the detailed reference values for kidney length ± 2 SD for each age and body height group. Since there was only one patient (two kidneys) with a body height less than 50 cm, they were excluded from Table 3. Tables 4 and 5 show the simple reference values for kidney length, regardless of sex or kidney position, for each age and body height group.

**Table 2:** Detailed reference values of kidney length by ultrasonography according to age

Age	(m/y)	Kidney length (cm)													
		Boys							Girls						
		Right kidney				Left kidney			Right kidney				Left kidney		
		*n*	Mean	Mean + 2sd	Mean -2sd	Mean	Mean + 2sd	Mean -2sd	*n*	Mean	Mean + 2sd	Mean -2sd	Mean	Mean + 2sd	Mean -2sd
0–2	(m)	21	5.0	6.2	3.8	5.1	6.0	4.2	7	4.9	6.0	3.7	5.2	6.5	3.9
3–5		25	5.5	6.6	4.4	5.6	6.6	4.6	6	4.9	6.5	3.3	5.3	7.1	3.6
6–8		17	5.4	6.7	4.1	5.8	7.0	4.5	4	5.2	7.4	3.0	5.5	7.0	4.1
9–11		17	5.5	6.7	4.3	5.8	6.6	4.9	9	5.7	6.8	4.6	6.0	7.2	4.7
12–17		23	5.8	6.5	5.2	6.0	7.0	4.9	13	5.7	6.8	4.6	6.1	7.1	5.0
18–23		12	5.9	7.1	4.7	6.4	7.5	5.3	6	6.3	7.3	5.3	6.4	7.8	4.9
2	(y)	20	6.5	7.5	5.5	6.7	7.6	5.8	26	6.4	7.4	5.4	6.6	7.4	5.7
3		108	6.6	7.7	5.6	6.9	8.0	5.8	174	6.7	7.8	5.6	6.9	8.0	5.8
4		44	7.1	8.3	5.9	7.3	8.9	5.7	65	7.0	8.2	5.9	7.2	8.5	6.0
5		46	7.4	8.6	6.3	7.6	8.9	6.4	46	7.5	8.7	6.3	7.5	8.8	6.1
6		78	7.7	9.0	6.4	7.8	9.2	6.4	86	7.8	9.0	6.6	7.9	9.3	6.5
7		65	7.9	9.3	6.6	8.1	9.7	6.5	96	8.0	9.3	6.7	8.1	9.6	6.6
8		59	8.1	9.3	6.9	8.3	9.6	6.9	81	8.3	9.7	7.0	8.5	9.9	7.0
9		43	8.3	9.5	7.0	8.5	9.8	7.3	66	8.2	9.7	6.8	8.5	10.1	6.8
10		45	8.7	10.0	7.4	9.0	10.3	7.7	74	9.0	10.5	7.6	9.2	10.7	7.7
11		46	9.2	10.6	7.8	9.3	10.9	7.7	94	9.4	10.9	8.0	9.5	10.9	8.1
12		72	9.6	11.6	7.7	9.7	11.6	7.8	85	9.7	11.2	8.2	9.9	11.5	8.3
13		55	10.2	11.9	8.5	10.4	11.9	9.0	71	9.9	11.5	8.3	10.0	11.5	8.4
14		55	10.0	11.4	8.6	10.2	11.8	8.6	60	10.0	11.3	8.6	10.2	11.6	8.7
15		30	10.4	12.3	8.5	10.5	12.3	8.6	17	10.0	11.6	8.5	10.2	11.5	8.8
16		4	10.4	12.6	8.3	10.3	12.8	7.8	4	10.2	12.3	8.2	10.5	14.2	6.7
17		2	11.0	11.1	11.0	11.3	11.6	11.0	2	10.1	10.1	10.1	10.6	11.0	10.1
18		2	10.2	10.3	10.0	10.3	10.4	10.3	3	9.8	10.4	9.2	10.2	12.1	8.3

*SD* standard deviation, *m* months, *y* years

**Table 3:** Detailed reference values of kidney length by ultrasonography according to body height

Body height (cm)	Kidney length (cm)													
	Boys							Girls						
	Right kidney				Left kidney			Right kidney				Left kidney		
	*n*	Mean	Mean + 2sd	Mean -2sd	Mean	Mean + 2sd	Mean -2sd	*n*	Mean	Mean + 2sd	Mean -2sd	Mean	Mean + 2sd	Mean -2sd
50–59.9	12	5.0	6.4	3.5	5.0	5.9	4.2	6	4.6	5.4	3.9	4.9	5.3	4.6
60–69.9	20	5.3	6.4	4.3	5.5	6.5	4.5	11	5.3	6.9	3.6	5.4	6.2	4.5
70–79.9	27	5.7	6.9	4.6	5.9	7.0	4.7	18	5.9	6.9	4.9	6.2	7.0	5.4
80–89.9	25	6.3	7.5	5.2	6.6	7.5	5.7	40	6.4	7.5	5.2	6.5	7.3	5.6
90–99.9	90	6.6	7.7	5.5	6.9	7.9	5.8	170	6.7	7.7	5.8	6.9	8.0	5.8
100–109.9	64	7.2	8.2	6.1	7.4	8.7	6.0	80	7.3	8.5	6.1	7.4	8.8	6.1
110–119.9	93	7.7	9.0	6.5	7.8	9.1	6.5	116	7.8	8.9	6.7	7.8	9.2	6.5
120–129.9	85	8.1	9.3	6.9	8.2	9.5	6.9	128	8.2	9.2	7.1	8.3	9.4	7.2
130–139.9	77	8.5	9.7	7.2	8.8	10.1	7.5	98	8.6	10.2	7.1	8.7	10.3	7.1
140–149.9	46	9.2	10.7	7.7	9.3	10.8	7.8	130	9.3	10.4	8.1	9.4	10.9	7.9
150–159.9	71	9.5	11.1	8.0	9.8	11.5	8.1	166	9.9	11.1	8.6	10.0	11.3	8.7
160–169.9	93	10.1	11.4	8.7	10.3	11.7	8.9	58	10.2	11.9	8.6	10.3	12.0	8.6
170–179.9	40	10.6	11.9	9.3	10.6	11.9	9.3							
180–189.9	5	11.4	14.3	8.4	11.4	12.8	10.1							

*SD* standard deviation

**Table 4:** Simple and practical reference values of kidney length by ultrasonography for practical clinical use by age

Age	(m/y)	*n*	kidney length (cm)		
			mean	mean + 2SD	mean -2SD
0–2	(m)	56	5.0	6.1	3.9
3–5		62	5.4	6.7	4.2
6–8		42	5.5	6.9	4.2
9–11		52	5.7	6.8	4.6
12–17		72	5.9	6.9	4.9
18–23		36	6.2	7.4	5.0
2	(y)	92	6.5	7.5	5.6
3		564	6.8	7.9	5.7
4		218	7.2	8.5	5.8
5		184	7.5	8.7	6.3
6		328	7.8	9.1	6.5
7		322	8.0	9.5	6.6
8		280	8.3	9.7	7.0
9		218	8.4	9.8	6.9
10		238	9.0	10.5	7.6
11		280	9.4	10.9	7.9
12		314	9.7	11.5	8.0
13		252	10.1	11.7	8.5
14		230	10.1	11.6	8.6
15		94	10.3	12.0	8.6
16		16	10.4	12.8	7.9
17		8	10.7	11.7	9.8
18		10	10.1	11.1	9.1

*SD* standard deviation, *m* months, *y* years

**Table 5:** Simple and practical reference values of kidney length by ultrasonography for practical clinical use by body height

Body height (cm)	*n*	kidney length (cm)		
		Mean	Mean + 2SD	Mean -2SD
50–59.9	36	4.9	5.9	3.9
60–69.9	62	5.4	6.5	4.3
70–79.9	90	5.9	7.0	4.8
80–89.9	130	6.4	7.4	5.4
90–99.9	520	6.8	7.8	5.7
100–109.9	288	7.3	8.6	6.1
110–119.9	418	7.8	9.0	6.5
120–129.9	426	8.2	9.4	7.0
130–139.9	350	8.6	10.1	7.2
140–149.9	352	9.3	10.7	7.9
150–159.9	474	9.9	11.3	8.4
160–169.9	302	10.2	11.7	8.7
170–179.9	82	10.6	12.0	9.2
180–189.9	10	11.4	13.6	9.2

*SD* standard deviation

### Formula for estimating kidney length by ultrasonography

As shown in Supplementary Fig. S1, the reference values of kidney length for each height were almost completely linear when plotted on the height-length graph, while those for each age formed a curve. From this, we decided to use height data for the prediction formula of kidney length.

**Fig. 2:**
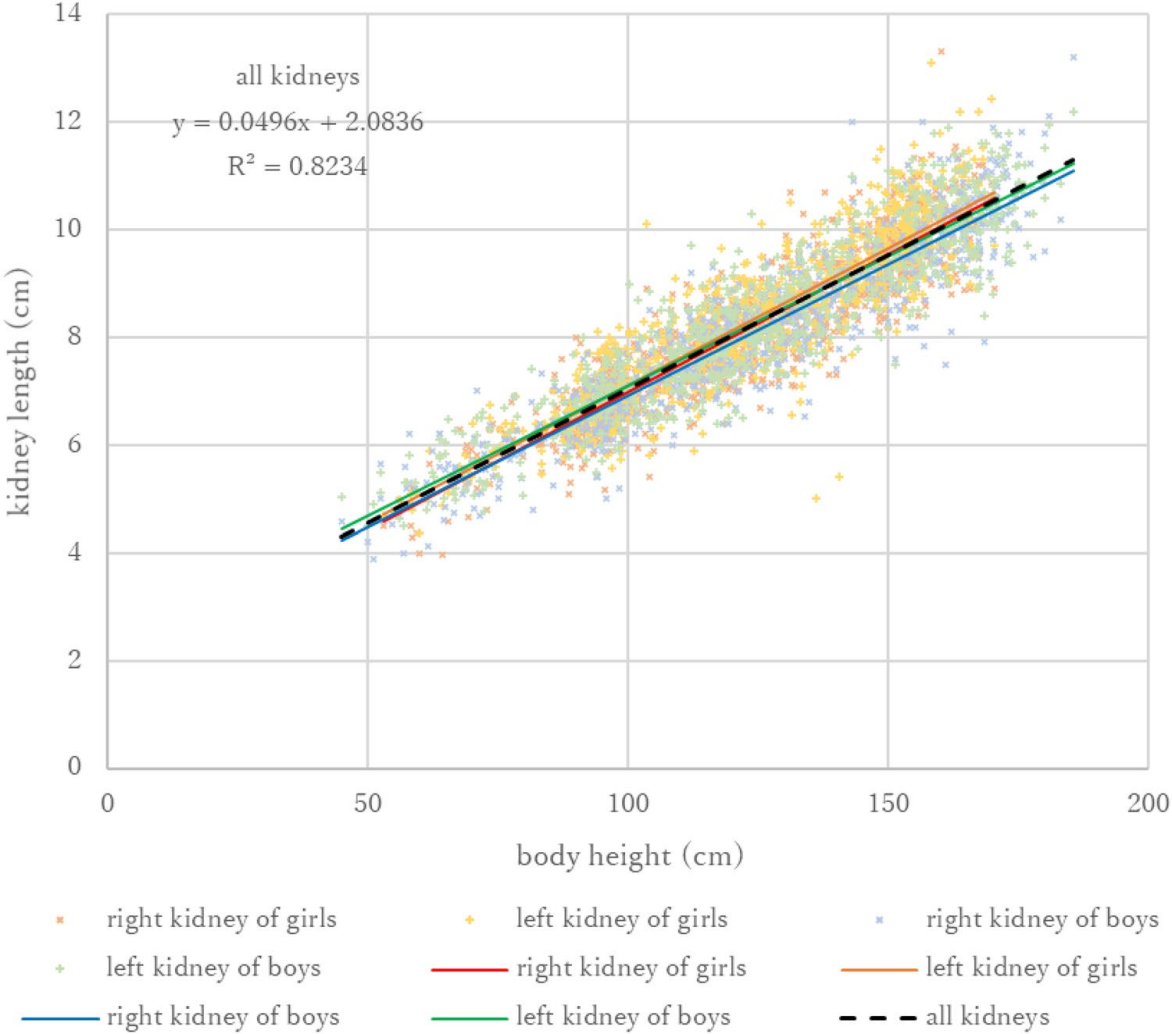
Kidney length by ultrasonography of right (light red cross) and left (light orange cross) kidneys of girls and those of right (light blue cross) and left (light green cross) kidneys of boys according to body height. Colored solid lines show each regression line, and the thick black dashed line indicates the regression line for all participants

The kidney lengths measured by ultrasonography by body height for 1771 participants (3542 kidneys) and their regression lines are shown in Fig. 2. The regression formula and coefficient of determination of kidney length (cm) and body height (cm) for all participants combined was *y* = 0.496x + 2.0836 (*R*^2^ = 0.8234).

### A simple yet practical formula for estimating average kidney length by ultrasonography

Based on the regression equation for all participants mentioned above, each term was rounded off to the first decimal place and as a result, we set a simple and practical formula for estimating the average value of kidney length by body height as “body height (m) × 5 + 2”. Supplementary Fig. S2 shows the measured ultrasonographic values of kidney length by body height, their regression line, and a straight line indicating the results of the estimated average values of kidney length calculated by this formula.

### A simple formula for estimating the lower limit of normal kidney length for children by ultrasonography

Table 6 shows the results of the “2 SD/mean” reference values for each height group. The calculated average value for “2 SD/mean” values from the data with a sufficient number of participants of 100 or more was 0.156, and the rounded down value of 0.15 was applied to “mean × (1–2 SD / mean)”. As a result, the formula for calculating the value of the lower limit of kidney length was set as “mean × 0.85” i.e. “0.85 × [body height (m) × 5 + 2]”.

**Table 6:** The 2 SD and “2 SD/mean” reference values for each height group, and the number and rate of subjects whose actual kidney length were judged shorter than the lower limit by the formula “estimated kidney length by body height × (1–0.15) (cm)”

Body height (cm)	*n*	2SD (cm)	2SD/mean	Number and rate of subjects lower than the value of “estimated kidney length by body height × (1–0.15) (cm)”	
				*n* (kidney)	Rate (%)
50–59.9	36	1.01	0.20	1	2.8
60–69.9	62	1.12	0.21	3	4.8
70–79.9	90	1.09	0.18	3	3.3
80–89.9	130	1.03	0.16	4	3.1
90–99.9	520	1.05	0.15	9	1.7
100–109.9	288	1.27	0.17	9	3.1
110–119.9	418	1.24	0.16	11	2.6
120–129.9	426	1.16	0.14	6	1.4
130–139.9	350	1.46	0.17	13	3.7
140–149.9	352	1.40	0.15	5	1.4
150–159.9	474	1.43	0.14	6	1.3
160–169.9	302	1.51	0.15	4	1.3
170–179.9	82	1.36	0.13	2	2.4
180–189.9	10	2.16	0.19	0	0.0

*SD* standard deviation

**Fig. 3:**
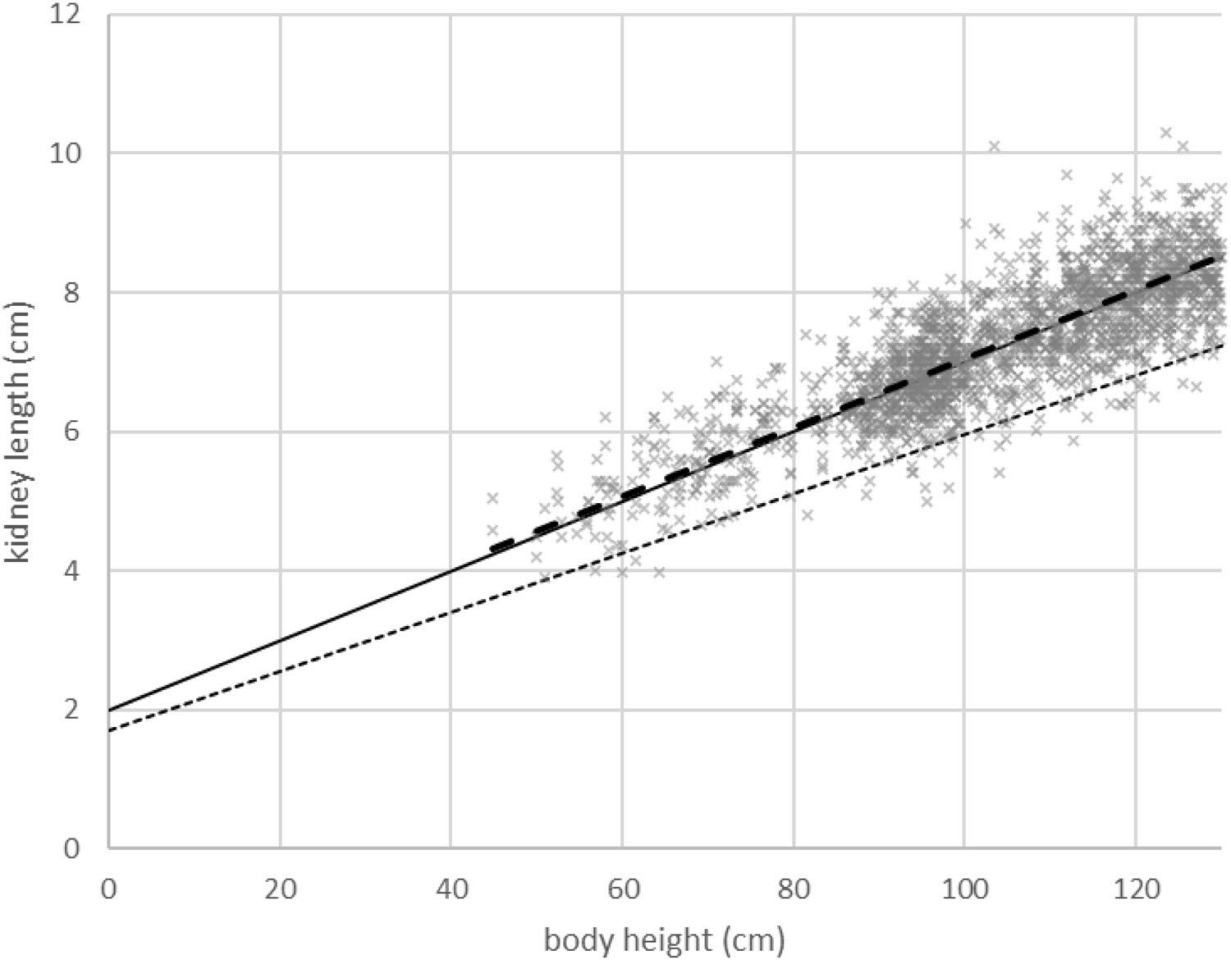
The measured values of kidney length by height by ultrasonography, their regression line (thick dashed line), and the estimated normal values of kidney length (solid line). The straight dotted line shows the lower limit of the normal range by this calculation formula (dotted line). Body height is shown in meters up to 1.3 m

The number and rate of participants whose actual kidney lengths were shorter than the lower limit by this formula is shown in Table 6. When the formula was applied to all cases or cases with a height of 130 cm or less, the rate of cases judged to be below the normal range was approximately 2.1% and 2.3%, respectively. The rate of being below the lower limit of the normal range tended to be small in participants with a body height of 140 cm or more. Figure 3 shows the measured kidney length by height, the estimated normal values of kidney length by height, and the lower limit of the normal range by this formula for children with body height up to 130 cm.

### Difference in measured values for each facility

**Fig. 4:**
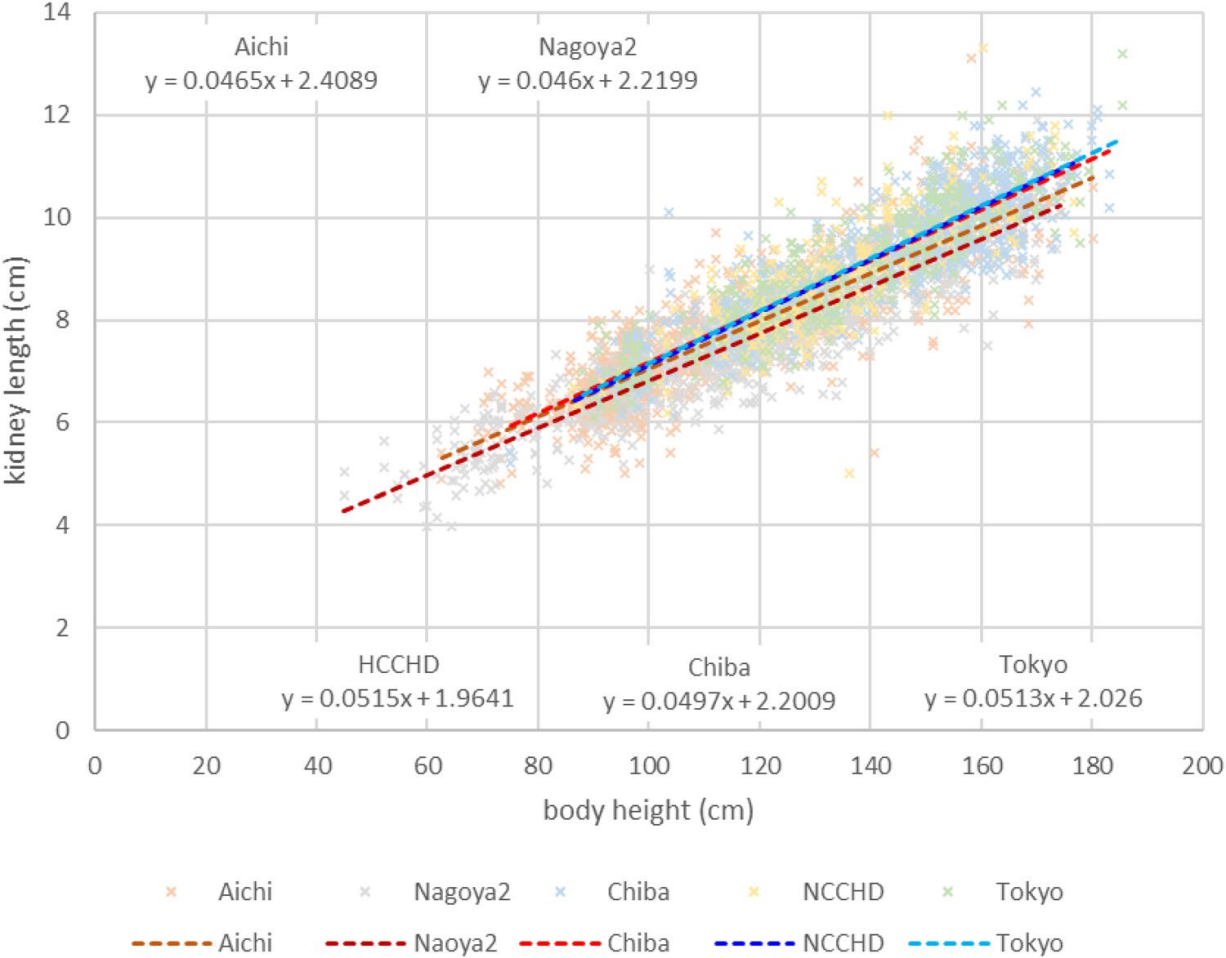
Kidney length by ultrasonography by body height for five facilities, which reported more than 100 cases. Aichi: Aichi Children’s Health and Medical Center, Nagoya2: Japanese Red Cross Aichi Medical Center Nagoya Daini Hospital, Chiba: National Hospital Organization Chibahigashi National Hospital, NCCHD: National Center for Child Health and Development, Tokyo: Tokyo Metropolitan Children’s Medical Center

Figure 4 shows the measured values for five facilities, which reported more than 100 cases. As shown in the figure, there were differences in the measured values depending on the facility.

### Differences in measured values according to sex, kidney position, and body position

The regression lines, regression formulas, and coefficients of determination for boys and girls, for right and left kidneys, and for each body position are shown in Supplementary Fig. S3 and S4. The regression lines for body height and kidney length in all participants along with their 95% confidence intervals are also shown in Supplementary Fig. S5 and S6. Each regression line was within a narrow range, but some segments of the lines were found to deviate from the 95% confidence intervals.

## Discussion

In this study, we developed the ultrasonographic reference values for kidney length in Japanese children based on the data of 1984 participants (3968 kidneys) from eight facilities throughout Japan. Detailed and simple reference value tables by age and by height were prepared; the detailed tables were shown separately by sex, and by right or left position, and the simple tables were shown regardless of sex or position. Additionally, we developed an estimation formula for kidney length by ultrasonography. The regression equation of kidney length (cm) and body height (cm) was “*y* = 0.0496x + 2.0836 (*R*^2^ = 0.8234)”. Based on these results, we proposed the following simple and practical estimation formulas: “the estimated average kidney length (cm) = body height (m) × 5 + 2” for all children, and “the estimated lower limit of normal kidney length (cm) = 0.85 × [body height (m) × 5 + 2]” for children up to 130 cm tall. These are the first reference values created with a sufficiently large number of participants, and simple reference values and estimating formulas are expected to be widely utilized in daily clinical practice.

Reliable reference values for clinical use should be based on the data of a sufficient number of participants. However, most previous reports on ultrasonographic reference values of kidney length in children were based on the data of approximately 200 to 1000 participants, and the number of participants per age group was small [3, 4, 6]. Our reference values, especially the simple ones, were considered more reliable than previously reported values because they were based on a much larger number of participants for each age and height group.

The clinical reference values should be applicable to a variety of situations at multiple institutions. Most previous reports of kidney size by ultrasonography in children were based on measurements performed by a limited number of examiners on the same ultrasonography device in the same body position and in a single facility [5]. However, it has not been fully verified whether these values can be applied when other examiners use different models at other facilities or with different body positions. The data we used to create reference values in this study were obtained from multiple examiners at eight facilities throughout Japan using different ultrasonography systems. We also did not limit the body position at the time of ultrasonography. Previous reports have shown that kidney length differs depending on the body position during ultrasonography [8]. Although we could not analyze kidney length in the lateral and sitting positions due to the insufficient number of participants, we did find that the difference between the supine and prone positions was small and practically negligible (Supplementary Fig. S4 and S6). Therefore, our results can be considered to be more widely applicable than previously reported findings and may be specifically used for measurements in the prone and supine positions.

There may be differences in kidney length depending on gestational age and birth weight [9]. Given that considerable data on gestational age or birth weight were lacking among the participants included in this study (Table 1), it should be noted that our results include findings of cases wherein where we could not clearly ascertain preterm birth and/or of participants with a low birth weight history. As a reference, data on kidney length in participants with clear data on gestational age and body weight at birth are shown in Supplementary Fig. S7.

Simple reference values can be more useful for daily clinical use than detailed ones. However, the creation of simple reference values by combining the data of both left and right kidneys could be considered controversial, because the length of the left kidney has been reported to be slightly longer than the right kidney in previous studies [6, 10]. In this study, slight but significant differences in length were also observed between the left and right kidney, and between boys and girls (Fig. 2, Supplementary Fig. S1, S3, and S5). However, these differences were considered minor enough to be practically insignificant in daily clinical practice. Therefore, we thought that the simple reference values that we developed by combining all the data can be applied to daily clinical practice regardless of sex and kidney position.

A simple predictive formula that can easily estimate normal kidney length at the bedside would be highly useful in clinical practice. Several formulas for predicting the normal length of kidneys in children based on age and/or body height have been shown in previous reports (Supplementary Table S1) [3–6, 10–13]. However, these formulas were relatively complicated for daily use at the bedside. Therefore, we tried to create a simpler estimation formula. Of the formulas shown in Supplementary Table S1, there were similarities between the formulas of Kim et al. [11] and our own. This could be due to the fact that both sets of formulas were based on data from East Asian children.

Whether age or body height is more appropriate as a parameter for estimating kidney length has not been sufficiently investigated. One previous study reported a simple formula that used age data to predict normal kidney length in children [6]. However, in our data on kidney length, the relationship with body height was almost linear, while the relationship with age was a curve similar to a growth curve. Furthermore, although there was almost no difference in kidney length by body height between boys and girls, kidney length tended to differ by age between boys and girls after puberty (Supplementary Fig. S1). Therefore, we considered body height a more appropriate measure than age to establish a single, simple, and practical predictive formula. For these reasons, based on the regression equation of body height and kidney length from our results, we propose “estimated average kidney length (cm) = body height (m) × 5 + 2” as a simple prediction formula. Using this formula, the estimated average kidney length can be easily calculated at the bedside regardless of sex and kidney position.

The normal range of kidney length is as important as the average value, and it would be highly useful if the lower limit of normal kidney length can be easily clarified at the bedside. Therefore, we suggest that “0.85 × the estimated average value of kidney length (cm).” could be used as the prediction formula for estimating the lower limit of kidney length.

However, when this formula was applied to participants with a body height of 140 cm or more, the rate of being judged below the lower limit was low (Table 6); hence, it was considered inappropriate to apply this formula to taller participants. In contrast, when this formula was applied to those with a body height of 130 cm or less, which corresponds to under 10 years of age, 2.3% were judged to be below the normal range (Table 6); therefore, it was considered appropriate for this prediction formula to be used for children with a body height of 130 cm or less.

There are some limitations to this study. First, we included patients with asymptomatic hematuria, benign familial hematuria, and monosymptomatic nocturnal enuresis, in addition to healthy children who underwent ultrasonography during physical examination. Strictly speaking, these patients may not be considered healthy. However, we only excluded children with diseases that might affect kidney size. Second, some cases reported as SFU grade 1 were not described as bilateral or unilateral; therefore, the actual number of kidneys with SFU grade 1 was unknown. Third, the number of participants varied depending on the age and body height categories. For example, the number of 3-years-old children included was extremely large compared to other age groups; both younger and older, due to the medical examination required of 3-years-old children in Japan. Since the number of participants under 2 and over 16 years was insufficient, as well as those with body height less than 60 cm or over 180 cm, the reference values for kidney length in these age and body height groups were unreliable. Finally, we conducted research by collecting data measured by multiple examiners using different types of ultrasound machine systems at multiple facilities throughout Japan. The technical quality of each examiner who performed ultrasonography was guaranteed based on the study method that specified the qualification of the examiner. However, the differences in the measurement values due to technical skill variations of each examiner and ultrasound machine system distinctions were not examined.

## Conclusions

Ultrasonographic reference values and simple prediction formulas for normal kidney length in healthy Japanese children under 18 years were developed in this study. These reference values and prediction formulas can be applied in any facility regardless of sex, kidney position, presence of SFU grade 1 hydronephrosis, and body position at the time of ultrasonography. We propose “the estimated average kidney length (cm) = body height (m) × 5 + 2” as a simple and practical calculation formula for predicting normal kidney length in children under 18 years. We also propose the formula “the estimated lower limit of normal kidney length (cm) = 0.85 × [body height (m) × 5 + 2]” to estimate the lower limit of normal kidney length for children up to 130 cm tall.

## Supplementary Information

Below is the link to the electronic supplementary material.

Supplementary file1 (DOCX 719 kb)
