# Supplementary material for: Ultrasonographic reference values and a simple yet practical formula for estimating average kidney length in Japanese children
Source: Clin Exp Nephrol. 2022 Apr 16;26(8):808–18. doi: 10.1007/s10157-022-02205-0 (PMC9287225; doi:10.1007/s10157-022-02205-0)
Supplement: Supplementary file 1 — Supplementary file1 (DOCX 719 kb) [file 10157_2022_2205_MOESM1_ESM.docx]

**Electronic Supplementary Material**

**Clinical and Experimental Nephrology**

**Reference values and simple and practical formula for estimating average kidney length in Japanese children**

Naoya Fujita^1^, Osamu Uemura^2^, Ryoko Harada^3^, Chieko Matsumura^4^, Tomoyuki Sakai^5^, Yuko Hamasaki^6^, Koichi Kamei^7^, Kentaro Nishi^7^, Tetsuji Kaneko^8^, Kenji Ishikura^9^, Yoshimitsu Gotoh^10^

On behalf of the Pediatric CKD Study Group in Japan in conjunction with the Committee of Measures for Pediatric CKD of the Japanese Society of Pediatric Nephrology

1. Department of Pediatric Nephrology, Aichi Children’s Health and Medical Center, 426 7-chome, Morioka-cho, Obu, Aichi 474-8710 Japan

2. Department of Pediatrics, Ichinomiya Medical Treatment & Habilitation Center, 1679-2 Tomida-nagaresuji, Ichinomiya-city, Aichi, 494-0018, Japan

3. Department of Nephrology, Tokyo Metropolitan Children's Medical Center, 2-8-29 Musashidai, Fuchu, Tokyo, 183-8561 Japan

4. Department of Pediatrics, National Hospital Organization Chibahigashi National Hospital, 673 Nitonacho, Chuo-ku, Chiba-city, Chiba, 260-8712, Japan

5. Department of Pediatrics, Shiga University of Medical Science, Seta, Otsu, Shiga, 520-2192, Japan

6. Department of Nephrology, Toho University Faculty of Medicine, 6-11-1 Omori Nishi, Ota-ku, Tokyo, 143-8541, Japan

7. Division of Nephrology and Rheumatology, National Center for Child Health and Development, 2-10-1 Okura, Setagaya-ku, Tokyo, 157-8535, Japan

8. Division of Clinical Research Support Center, Tokyo Metropolitan Children’s Medical Center, 2-8-29 Musashidai, Fuchu, Tokyo, 183-8561 Japan

9. Department of Pediatrics, Kitasato University School of Medicine, 1-15-1 Kitazato, Minami-Ku, Sagamihara, Kanagawa 252-0374 Japan

10. Department of Pediatric Nephrology, Japanese Red Cross Aichi Medical Center Nagoya Daini Hospital, 2‐9 Myoken‐cho Showa‐ku Nagoya‐shi Aichi 466‐8650 Japan.

The e-mail address, telephone and fax numbers of the corresponding author:

Naoya Fujita^1^

E-mail: fujita708@hkg.odn.ne.jp

**Supplementary Table S1**. Formulae for predicting the normal length of kidneys in children in previous reports

| Author | Formulae for predicting normal length |
| --- | --- |
| Duminda WD [10] | <16 months left kidney = 3.827 + 0.019(length in centimeters) + 0.141(weight in kilograms) - 0.023(age in months) - 0.347(for male sex) (R^2^=0.592) right kidney = 3.888 + 0.020(length or height) + 0.121(weight) - 0.037(age) - 0.372 (for male sex) (R^2^=0.535) 17< months left kidney = 5.651+ 0.022(age) + 0.01(BMI) (R^2^=0.625) right kidney = 5.336 + 0.022(age) + 0.012(BMI) (R^2^=0.661) |
| Oh MS [4] | right kidney length (cm)= 0.051 × height (cm) + 2.102 left kidney length (cm)= 0.051 × height (cm) + 2.280 |
| Kim JH [8] | Renal length (cm): Boys:  right = 2.270 + 0.046 × Height (± 1.200) left =2.203 + 0.048 × Height (± 1.192) Girls:  right = 2.485 + 0.044 × Height (± 1.072) left = 2.528+ 0.045 × Height (± 1.152) All groups:  right = 2.383 + 0.045 × Height (± 1.135) left= 2.374 + 0.047 × Height (± 1.173) |
| Otiv A [5] | Renal Length = 0.0421 × height + 2.6311 Renal Length = 0.3055 × age + 5.2533 |
| Akhavan A [6] | ＜1 year length (cm) = 0.159 × age (months)＋4.457（R^2^=0.533） ＞1 year length (cm) = 0.278 × age (years)＋6.103（R^2^=0.812） The simplified formulas ＜1 year length (cm) = 0.2 × age (months)＋4（R^2^=0.33） ＞1 year length (cm)＝0.3 × age (years)＋6（R^2^=0.81） |
| Rosenbaum DM [3] | >1 year renal length (cm) = 6.79 + 0.22 × age (years) (R^2^=0.7077) <1 year renal length (cm) = 4.98 + 0.155 × age (months) (R^2^=0.3437) |
| Haugstvedt S [9] | Kidney length [(right + left) / 2] (mm) Body surface area (m2) y = 36.1x + 51.0 (r = 0.94) Body weight (kg) y = 0.95x + 59.7 (r = 0.91) Body height (cm) y = 0.42x + 33.7 (r = 0.93) Age (years) y = 3.1x + 60.6 (r = 0.90) |
| Hodson CJ [12] | Age/Kidney length 5-13 years. Length(cm) = 0.397×age (year) + 6.65 (r=0.857) Body Height/Kidney Length children. Length(cm) = 0.145 × height(inches) + 2.646(r=0.874) |

**Supplementary Fig. S1.**

**Fig. S1 (a)**


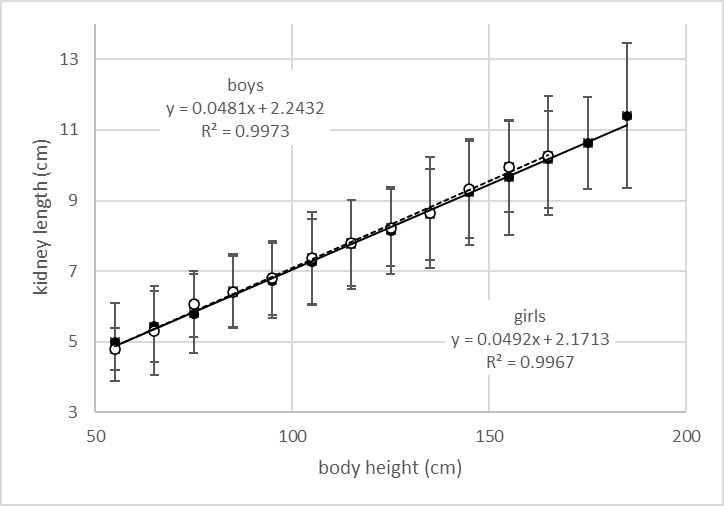


**Fig. S1 (b)**

**
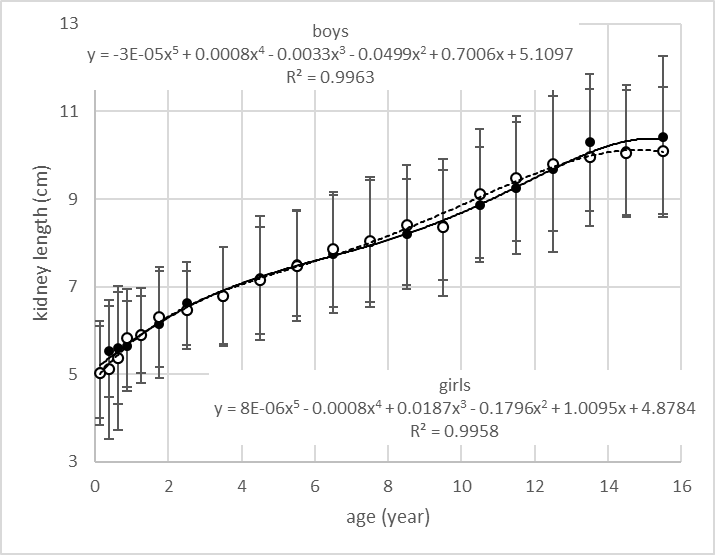
**

**Fig. S1 (a), (b)** The reference average values of kidney length for (a) each body height and (b) each age, by ultrasonography. The data are shown separately by sex, with the left and right kidney positions combined. Closed circles indicate data for boys, open circles indicate data for girls. The vertical bar indicates the range for ± 2SD. The solid line indicates the regression curve for boys, the dotted line indicates that for girls. Fig. S1(b) does not show data for age 16 or older, which had only few participants.

**Supplementary Fig. S2**


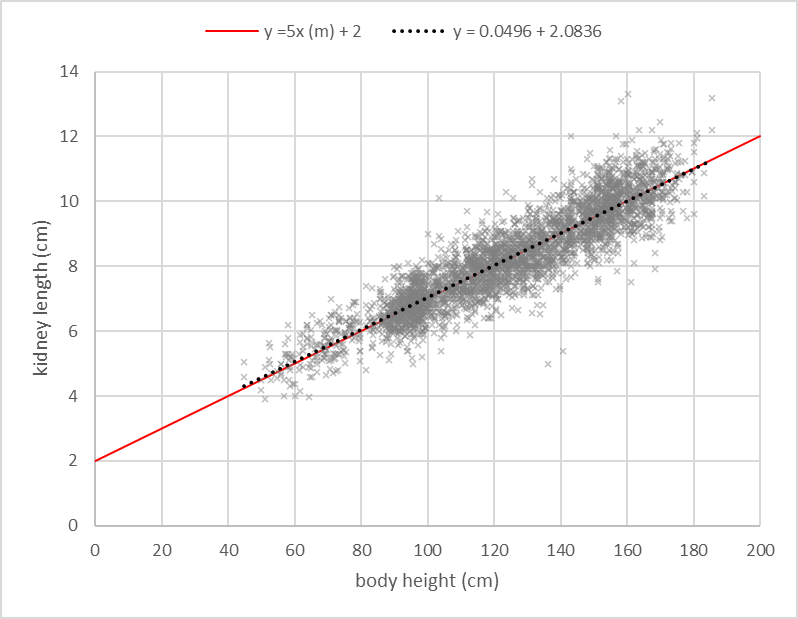


**Fig. S2.** The measured values of kidney length by ultrasonography according to body height and their regression line (*dotted black line*), and a straight line (*red solid line*) indicating the results of the estimated average values of kidney length calculated by this simple and practical prediction formula: “estimated average kidney length (cm) = body height (m) × 5 + 2”

**Supplementary Fig. S3.**

**Fig. S3 (a)**

**
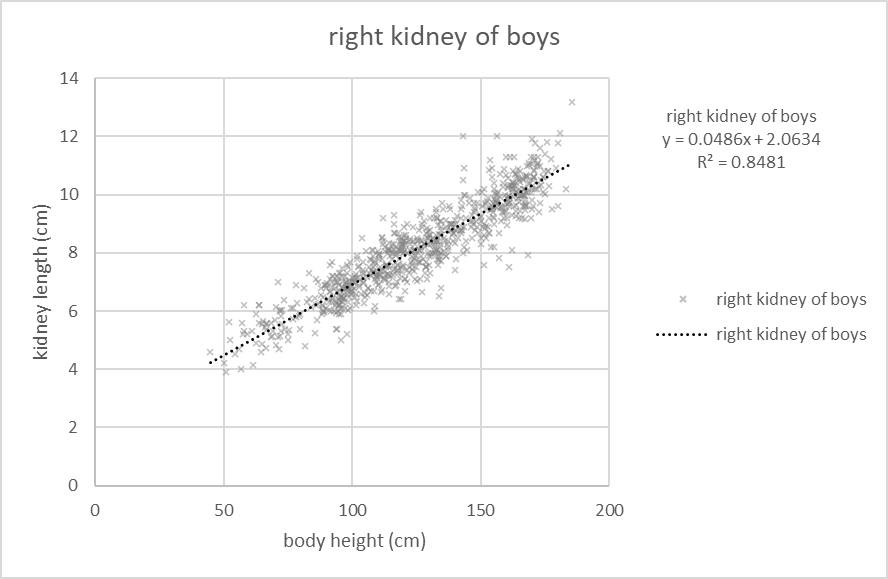
**

**Fig. S3 (b)**

**
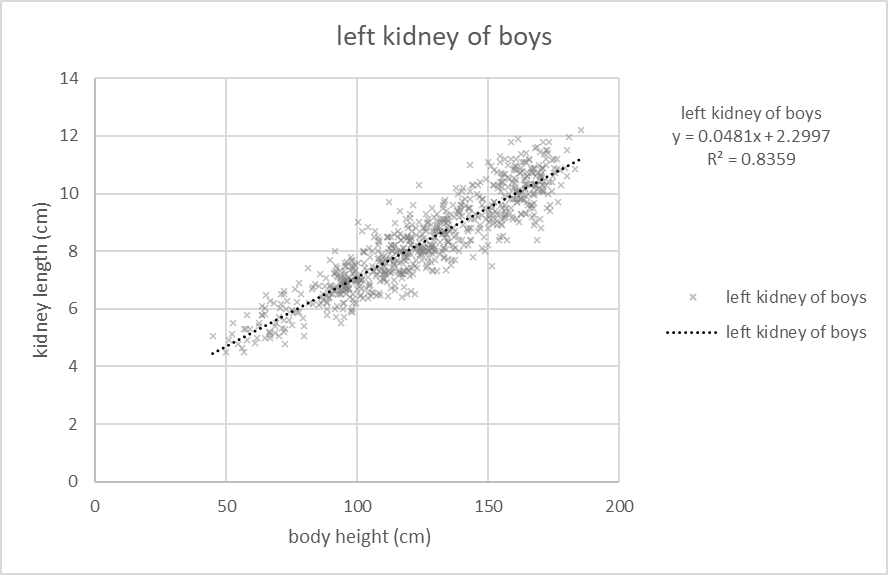
**

**Fig. S3 (c)**

**
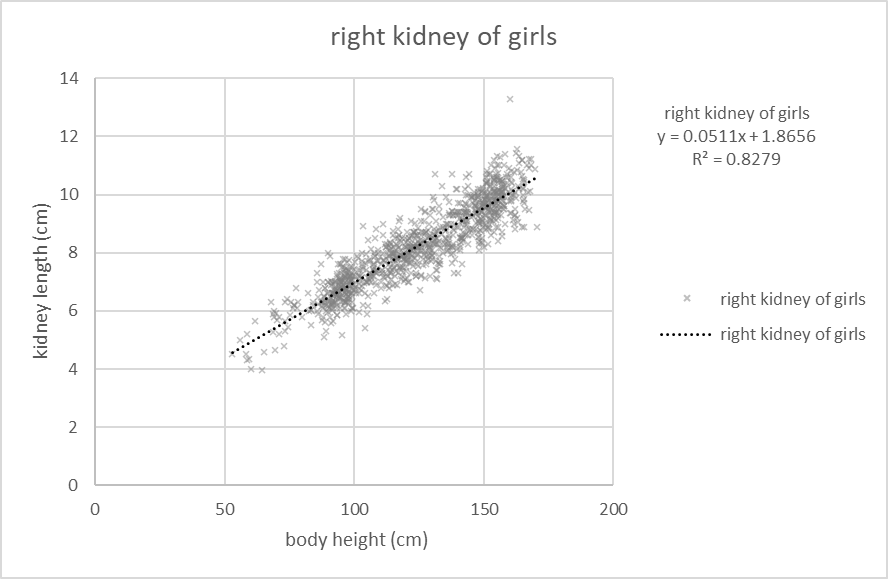
**

**Fig. S3 (d)**

**
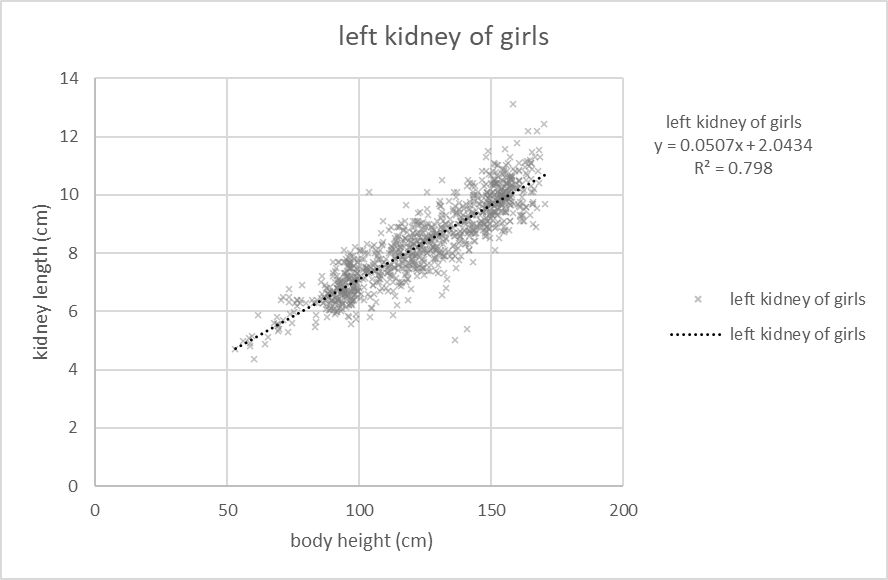
**

**Fig. S3.** Kidney length by body height, the regression lines (*dotted line*), the regression formulae and coefficient of determination for (a) right kidney of boys, (b) left kidney of boys, (c) right kidney of girls, and (d) left kidney of girls.

**Supplementary Fig. S4**


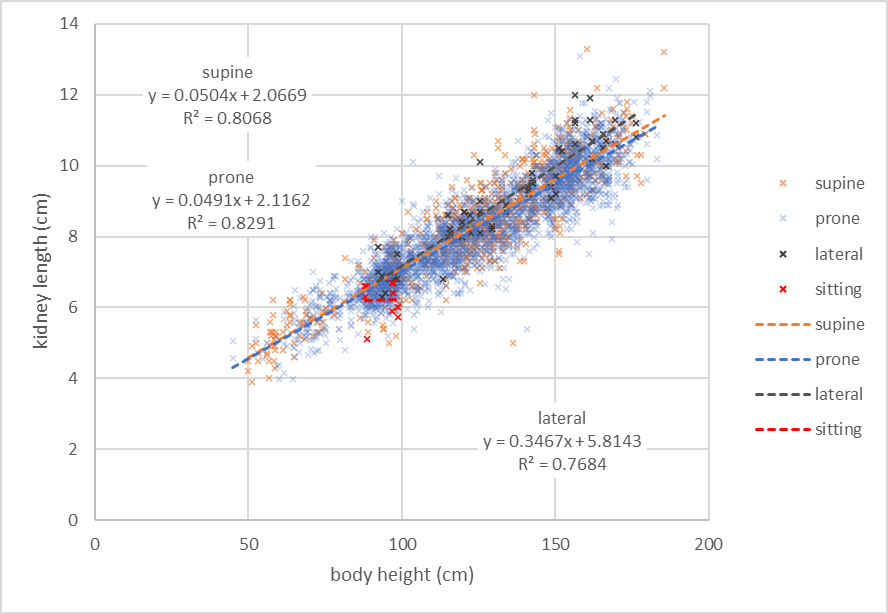


**Fig. S4.** The kidney length by ultrasonography according to body height in supine (*light orange cross*), prone (*light blue cross*), lateral (*black cross*), and sitting (*red cross*) position. The dotted line shows each regression line.

**Supplementary Fig. S5**

**
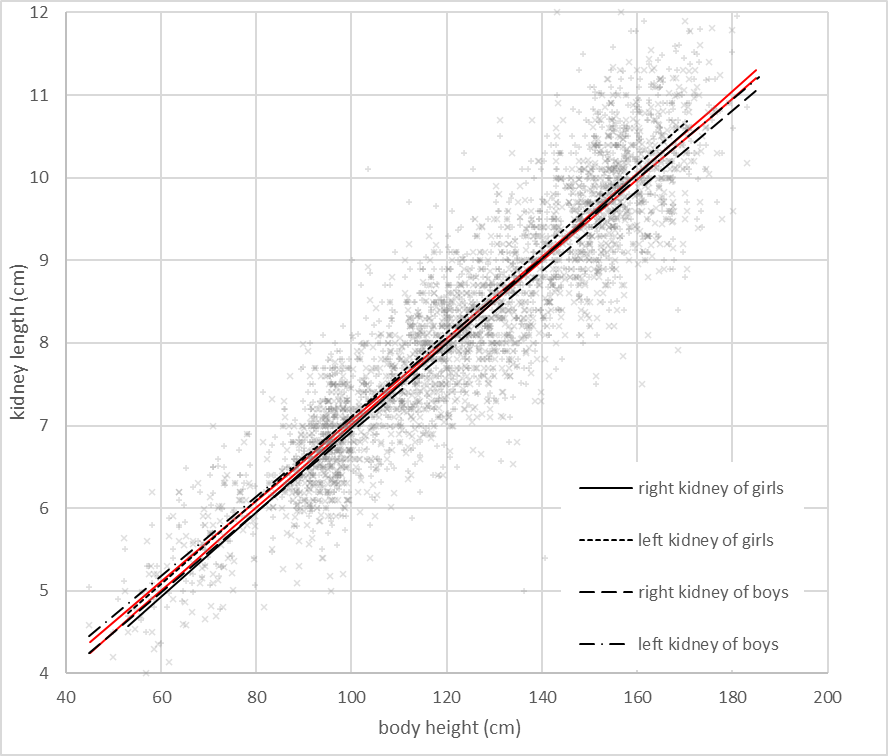
**

**Fig. S5.**

The upper and lower bounds of the 95% confidence interval for the regression line of kidney length and body height for all participants are shown by the solid red line. The black lines show the regression lines of kidney length and body height for right kidney of girls (solid line), left kidney of girls (dotted line), right kidney of boys (dashed line), and left kidney of boys (dashed-and-dotted line).

**Supplementary Fig. S6**


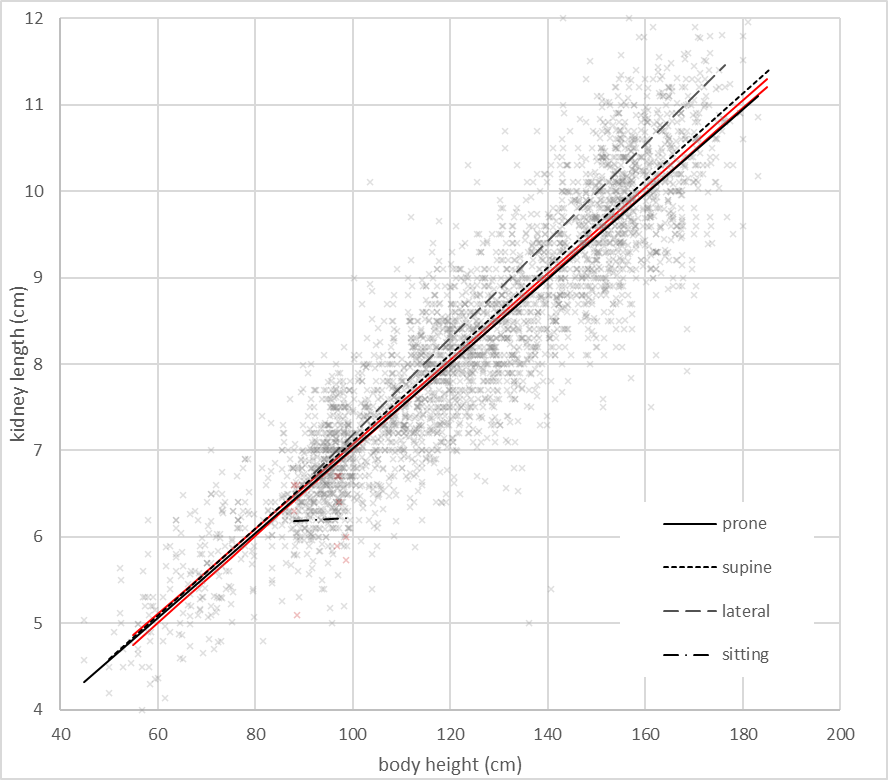


**Fig. S6.**

The upper and lower bounds of the 95% confidence interval for the regression line of kidney length and body height for all participants are shown by the solid red line. The black lines show the regression lines of kidney length and body height for prone (solid line), supine (dotted line), lateral (dashed line), and sitting position during ultrasonography (dashed-and-dotted line).

**Supplementary Fig. S7**

**Fig. S7(a)**

**
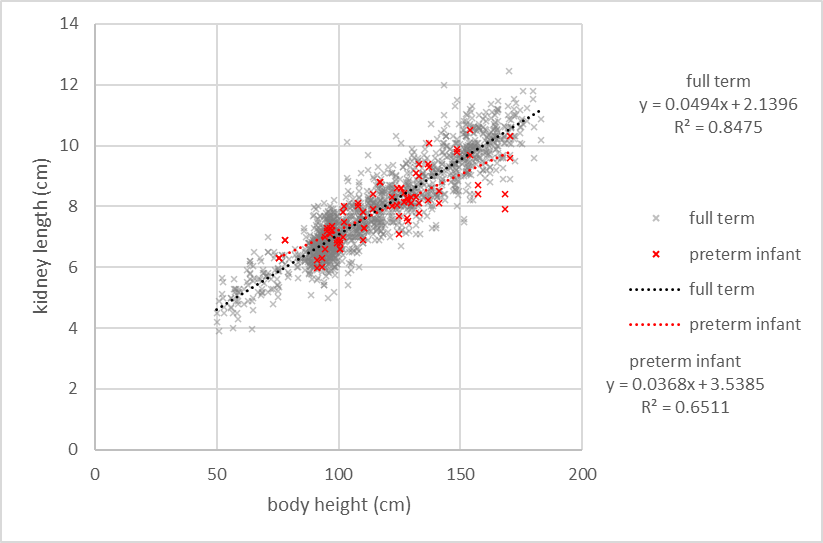
**

**Fig. S7 (b)**

**
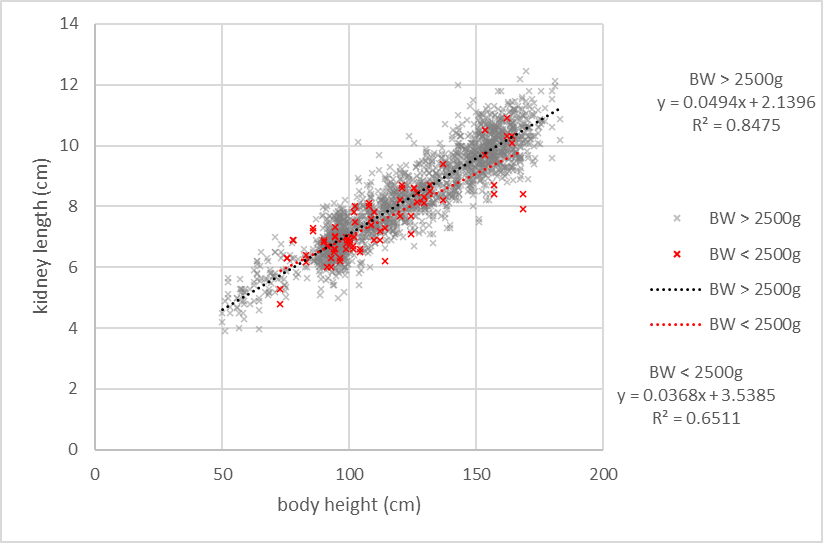
**

**Fig. S7.** The kidney length by ultrasonography according to body height for participants with (a) gestational age of 37 weeks or more (n = 1482) (*gray cross*) and under 37 weeks (n = 74) (*red cross*), and (b) birth body weight 2500 g (n = 2008) (*gray cross*) or more and less than 2500 g (n = 148) *(red cross*) at birth. The dotted line shows each regression line.
